# Cystic dystrophy of the duodenal wall in heterotopic pancreas: an atypical case in non-alcoholic female

**DOI:** 10.1259/bjrcr.20160058

**Published:** 2016-11-02

**Authors:** Marco Di Serafino, Rosa Severino, Raffaela Brigida, Enrico Scarano

**Affiliations:** ^1^Emergency Radiology Department, San Carlo Hospital, Potenza, Italy; ^2^Radiology Department, Federico II University Hospital, Napoli, Italy

## Abstract

Cystic dystrophy of the duodenal wall in heterotopic pancreas, recently described as paraduodenal pancreatitis, is a rare condition characterized by multiple cysts or pseudocysts located in the submucosa or muscularis propria of a thickened duodenal wall. They result from multiple episodes of obstruction of the small ducts in aberrant pancreatic islets. Cystic dystrophy of the duodenal wall in heterotopic pancreas usually affects alcoholic males, but here we present the case of a female without a history of alcohol abuse who was referred to our emergency department with abdominal pain and vomiting. She was treated by a pancreas-preserving surgical approach, thanks to a proper pre-operative differential diagnosis. Even though differentiating this benign condition from pancreatic cancer is a challenge, some characteristic findings on multidetector CT scan and MRI/MR cholangiopancreatography, such as a thickened duodenal wall containing cysts and sheet-like tissues in the pancreaticoduodenal groove, could lead to the correct diagnosis.

## Background

Cystic dystrophy of the duodenal wall in heterotopic pancreas (CDHP) is an uncommon complication of pancreatic heterotopia and is now widely known as paraduodenal pancreatitis.^1–3^ Its exact pathogenesis is still unknown but alcohol abuse is considered a precipitating factor.^1–7^ Consequently, this rare condition affects males more than females, alcoholics in particular.^2–3,5–7^ Usually, the lesions are recognized at autopsy^8^ while, when present, symptoms are non-specific, varying from vomiting, abdominal pain and jaundice.^2,4–8^ As in our case, imaging findings could be pivotal to achieving the correct diagnosis and excluding pancreatic malignancy to avoid useless and high-risk resection of the pancreas.

## Case report

A 59-year-old female presented to our emergency department with upper quadrant pain and vomiting. On physical examination, severe epigastric tenderness was present but the abdomen was not distended. The patient complained of weight loss of 5 kg and abdominal pain, which had progressively gotten worse over the previous 2 months, becoming continuous, increasing after meals and being relieved only after vomiting. There was no history of alcohol abuse. Blood sample test results revealed increase in amylase (156 U l^−1^; upper reference value 100) and cancer antigen 19-9 (52 U ml^−1^; upper reference value 37) levels, with normal complete blood counts.

## Investigations

**Figure 1.**
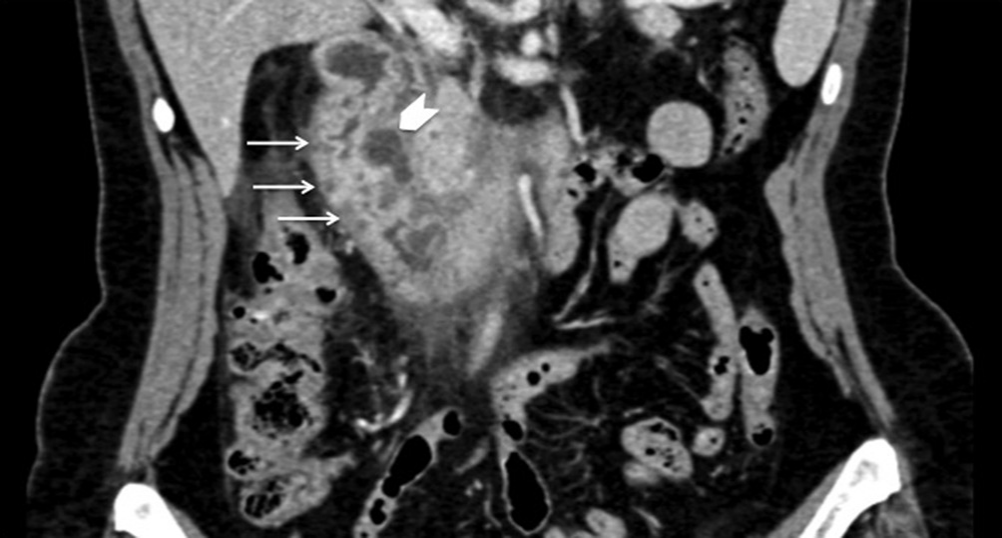
Multidetector CT, portal phase, coronal reconstruction shows hypoattenuating cystic lesion encircling the wall of the second part of the duodenum (arrowhead), with also circumferential wall thickening (arrows).

**Figure 2.**
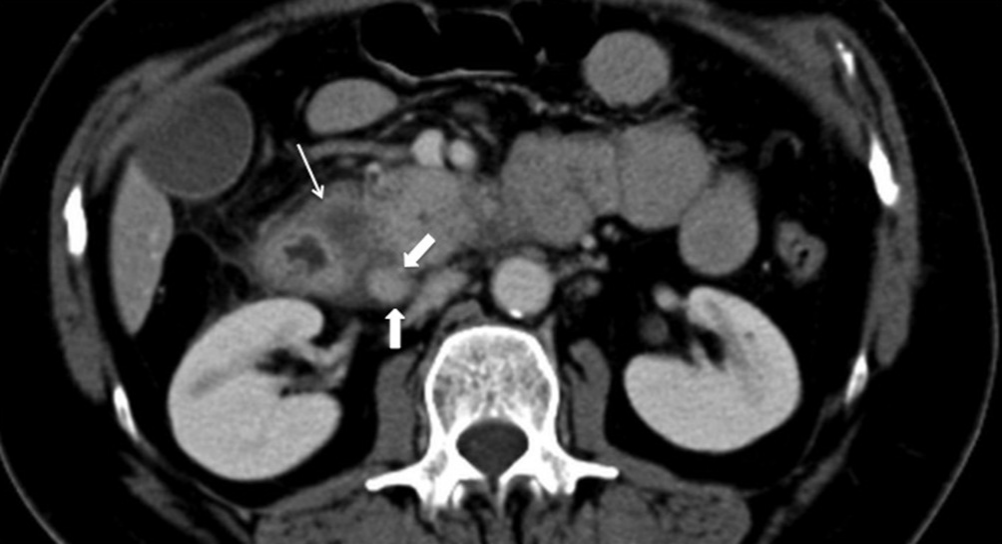
Mutidetector CT, portal phase, axial image shows thickening of the wall of the second portion of the duodenum, within which, on the pancreatic side, a cystic lesion can be appreciated (thin arrow); in addition, soft tissue almost isodense to the pancreas is seen between the pancreatic head and the duodenal wall (thick arrows).

**Figure 3.**
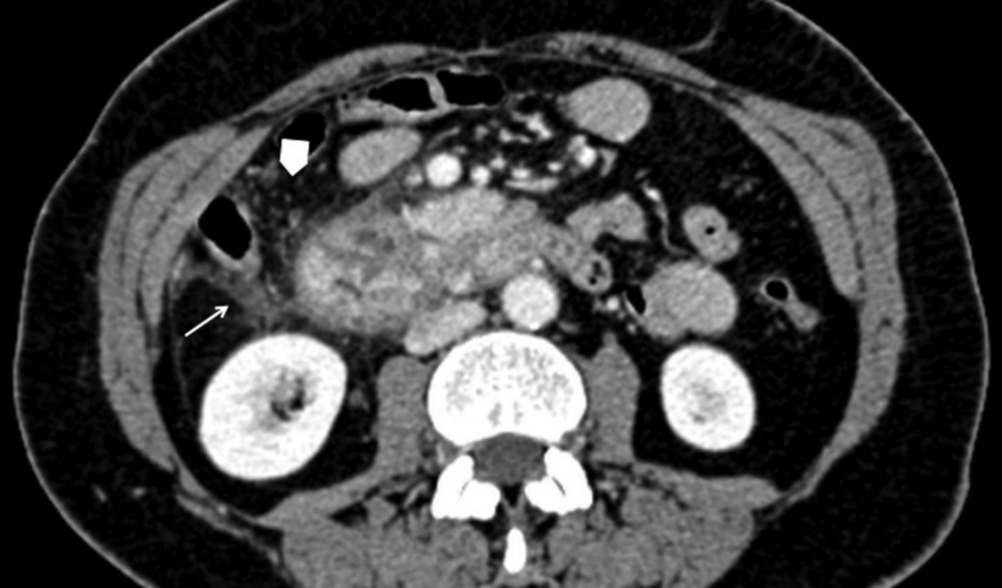
Multidetector CT, portal phase, axial image shows inflammatory changes involving the paraduodenal area with fluid (arrow) and fat thickening (arrowhead).

**Figure 4.**
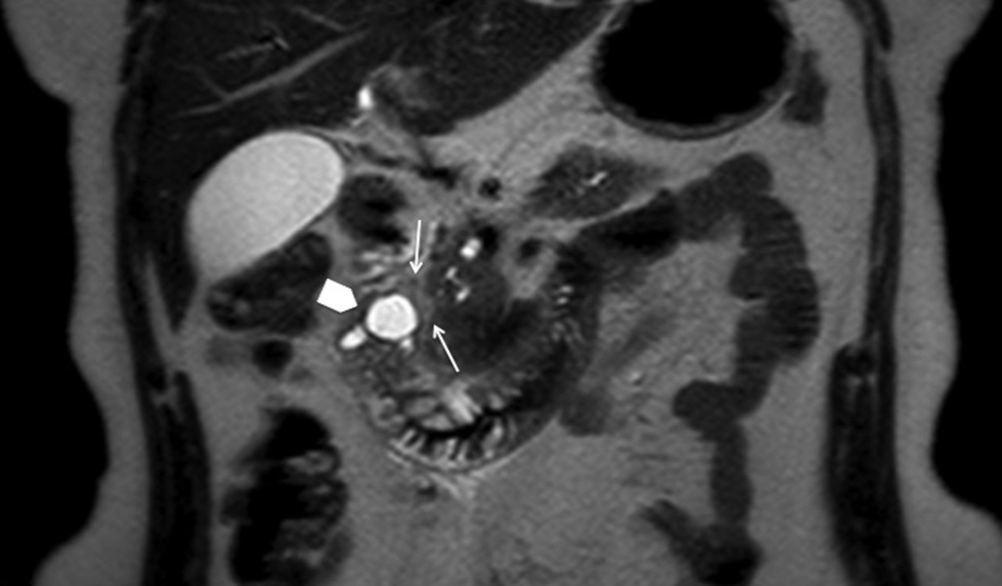
Coronal *T*_2_ weighted MRI shows intramural fluid-signal intensity lesions within the descending portion of the thickened duodenal wall (arrowhead) and sheet-like tissue adjacent to it (thin arrows), consistent with cystic dystrophy.

**Figure 5.**
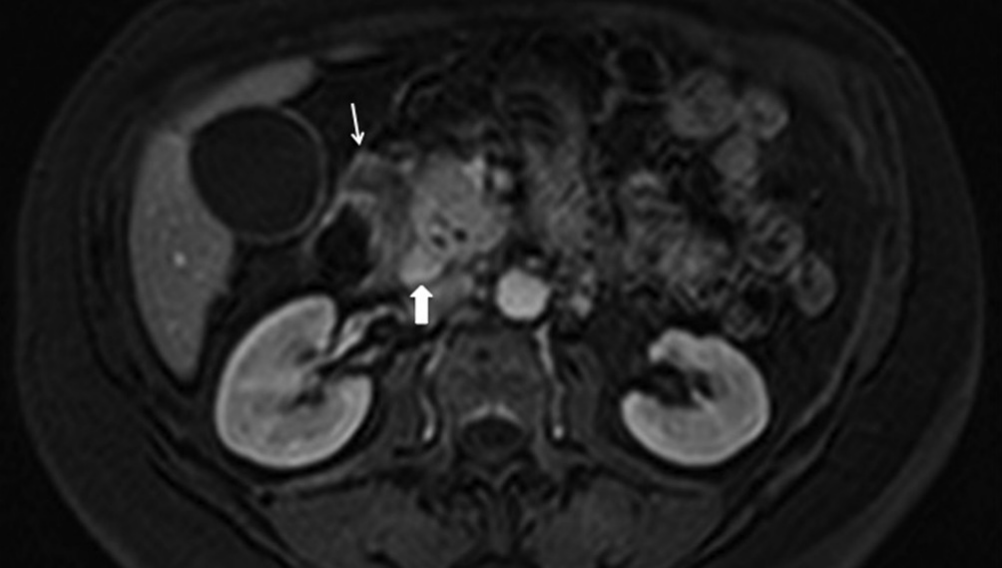
Axial *T*_1_ weighted MRI, portal phase shows pattern of enhancement of the sheet-like tissue in the pancreaticoduodenal groove, which is similar to the pancreas (thick arrow); within the duodenal wall there is a fluid-containing hypointense lesion (thin arrow).

On her first visit to our department, ultrasonographic examination did not show abnormalities of the abdominal parenchyma; her gallbladder was normal and there was no dilatation of the common bile duct (CBD). The patient also underwent an upper gastrointestinal endoscopy, which too was normal. Next, a multidetector CT (MDCT) scan was performed and revealed cystic lesions completely encircling the second portion of the duodenum (Figure 1). In addition, there was a solid component in the so-called pancreaticoduodenal groove, strictly contiguous to the pancreatic head, with heterogeneous enhancing pattern and areas isodense to the pancreas (Figure 2). Signs of inflammatory changes, such as fat thickening and fluid, surrounded the soft tissue in the pancreaticoduodenal area. Although these findings were suggestive of pancreatitis (Figure 3), the pancreatic head as well as the pancreatic duct appeared normal. However, to better characterize the solid component and its relationship with the pancreatic head and CBD, and to rule out a diagnosis of tumour, further examinations were requested. MRI (Figures 4–5) revealed the same MDCT scan findings, and an MR cholangiopancreatography confirmed normal gallbladder and no dilatation of the CBD or the pancreatic duct.

After first remission of the symptoms, a new MDCT scan was performed when the patient again experienced severe abdominal pain complicated by jaundice and it showed, in addition to the previous findings, markedly dilated intra- and extrahepatic biliary ducts.

## Differential diagnosis

The main and most important differential diagnosis of soft tissue mass in the groove area is groove pancreatic carcinoma. Although the absence of CBD narrowing may be seen in both malignant and benign conditions, being more frequent in the latter, the simultaneous presence of duodenal wall cysts is suggestive of CDHP. In addition, the pattern of enhancement, which is heterogeneous, with areas similar to the enhancing pancreas, is more typical of groove pancreatitis. On the contrary, adenocarcinoma is homogeneously hypoenhancing compared with the pancreas. Moreover, differentiating the duodenal cysts from duplication cysts is crucial to assess their location in the duodenal wall, which is also thickened.

## Treatment

A first attempt at medical treatment with analgesics and anti-inflammatory drugs was successful in relieving the clinical symptoms and the patient was discharged. After 2 months, the patient presented again with abdominal pain and jaundice and was then referred to the surgical team for removal of the aberrant tissue between the pancreatic head and the duodenum, and also the affected duodenal segment, with end-to-end anastomosis. The pancreatic head was spared.

## Outcome

On histopathological examination, heterotopic pancreatic tissue was identified within both the muscular layer of the duodenal wall and the solid component between the duodenum and the pancreatic head. These findings were suggestive of CDHP, with no elements of malignancy.

## Discussion

The presence of complete and mature pancreatic tissue outside its typical location and with no anatomical or vascular continuity with the pancreas itself is defined as heterotopic pancreas.^9^ The reported incidence of this rare condition varies between 1% and 14% in autopsy series.^8–9^ The lesions are mainly located in the duodenal (28%), jejunal (16%) or gastric (26%) walls, but may also be present in the biliary tract, and mesentery and umbilicus, or in the parenchymatous organs such as the liver and spleen.^9^

A rare complication of pancreatic heterotopia is a disease known as CDHP, which was first reported in 1970 by Potet and Duclert,^10^ the pathological features of which were defined by Fléjou et al^2^ in 1993.

However, the term currently used to denote this rare condition is paraduodenal pancreatitis, which unifies entities with shared clinicopathological features, including islets of pancreatic tissue in the duodenal wall, dilated ducts resulting in cysts and pseudocysts, Brunner’s gland hyperplasia, non-specific inflammation and fibrosis located in the “groove” of the duodenopancreatic region.^8^

Some authors have recently found that the presence, in the duodenal muscularis propria, of areas of pancreatic tissue could be an incomplete involution of dorsal pancreas rather than complete pancreatic heterotopia.^1^ Islets of aberrant pancreas seem to have an abnormal drainage in the duodenal wall near the minor papilla, which could contribute to obstruction of outflow in this area, especially if exposed to exogenous factors.^3^ As a consequence, accumulation of pancreatic secretions can lead to repeated episodes of obstructive acute pancreatitis and the formation of retention cysts, surrounded by inflammation and fibrosis.^1,4,11,12^ The fibrotic tissue that develops in the duodenal wall may also involve the groove between the wall and the pancreatic head, potentially resulting in compression and narrowing of the CBD. Microscopically, fibrosis is characterized by a chronic inflammatory process in the duodenal submucosa, which could involve the contiguous pancreatic tissue.^3^

The exact pathogenesis of the inflammatory changes is still unknown. Many authors argue that alcohol abuse could be a precipitating factor, as most of the patients with paraduodenal pancreatitis are alcoholics. However, the relative role of alcohol abuse or some anatomical anomalies of the minor papilla probably varies among patients, as could by deduced by the description of rare cases of similar cystic and inflammatory lesions that developed in gastric heterotopic pancreas, or in the absence of history of alcohol abuse.^1,3,9^

From a clinical point of view, the majority of patients are asymptomatic, with the lesions being incidentally detected during surgery or autopsy. The main symptoms are severe upper abdominal pain, post-prandial vomiting, nausea and weight loss owing to stenosis of the duodenum. Jaundice develops in approximately 20% of the patients.^2,4–8^ Chronic pancreatitis is usually a misdiagnosed complication, rarely recognized clinically or radiologically but only detected on microscopic analysis.^3,8^

On testing of blood samples, there is occasionally a slight elevation in pancreatic and, more rarely, hepatic enzymes.^1,5–6,8,13^

Most patients are relatively young (40–50 years old) males, with a history of heavy alcohol abuse.^2–7^ Less commonly, as in this case, the lesion can be observed in females (10–20% of cases) and non-alcoholic patients (10–20% of cases).^1^

Abdominal ultrasound is often the first-line imaging modality and may demonstrate a hypoechoic mass adjacent to the pancreatic head with thickening of the second part of the duodenum.^4^ Nevertheless, it is rarely diagnostic.^6^

On CT examination, the most specific finding is diffuse or eccentric thickening of the duodenal wall, which can lead to luminal narrowing and may be accompanied by cysts of various sizes and shapes in the duodenal wall and/or the groove area.^1,6–7,12^

The other common feature on CT scan is the hypo-isodense enhancing soft tissue mass in the pancreaticoduodenal groove, which is thought to represent inflammatory tissue.^4,6–8,14–15^ In a large series, Zaheer et al^5^ demonstrated this finding in all the cases, even if it could be detected as peripancreatic stranding around the pancreatic head or amass-like area. In addition, in 50% of patients there is also heterogeneity of the pancreatic head with a discrete hypoenhancing mass lesion, raising the suspicion of an underlying adenocarcinoma.

The same characteristic findings are seen on MRI. In particular, a sheet-like mass corresponding to the fibrous scar is recognized in the groove, showing different patterns of enhancement. Cystic lesions of the groove and/or the duodenal wall are well displayed on MRI and typically show hyperintense and hypointense signal on *T*_2_ and *T*_1_ weighted images, respectively.

Further evaluation with MR cholangiopancreatography is important to define the eventual stenosis of CBD, which is commonly smooth in paraduodenal pancreatitis and irregular in case of pancreatic cancer. Moreover, it may reveal widening of the space between the duodenal lumen, distal CBD and pancreatic ducts, with intramural and paraduodenal cysts.^4^ Differential diagnosis from pancreatic carcinoma is most difficult as well as most important.

In the case described above, diagnosis of paraduodenal pancreatitis was achieved after considering the duodenal features and the characteristic enhancement of soft tissue in the groove area.

Actually, both duodenal wall thickening and cystic changes are findings extremely uncommon in pancreatic adenocarcinoma.^5^ Moreover, even though the soft tissue mass in the pancreaticoduodenal groove could be misinterpreted as malignancy, particularly when it involves the pancreatic head, many authors suggest that patterns of enhancement, either progressively centripetal or “patchy”, are suggestive of groove pancreatitis.^4–8,15^

In the last few years, endoscopic ultrasound has emerged as the preferred imaging method as it readily demonstrates a hypoechoic area between the duodenal wall and the pancreatic parenchyma, thickening and narrowing of the duodenal lumen, associated pancreatic calcifications, pseudocysts, stenosis of the CBD and dilatation of the pancreatic duct.^4,6^ It is especially useful in the more accurate definition of duodenal wall layers, allowing differential diagnosis from duodenal duplication, necrotic duodenal tumours, pancreatic pseudocysts or cystic tumours.^11–12^

Despite both medical and surgical approaches being described in the literature, when there is no response to abstention from alcohol intake, analgesics and octreotide, endoscopic drainage of minor papilla or surgical treatment is needed.^5^ Duodenopancreatectomy seems to definitely remove the risk of recurrence and the potential impact on the pancreas.^4,7,11^

However, limited resection of the second part of the duodenum without pancreatectomy, a procedure which depends on the suprapapillary localization of the cystic lesions in the absence of significant changes in the pancreatic gland, demonstrated the same good results but with less associated morbidity and mortality.^13^

This targeted approach is possible, as in the present case, when the diagnostic flow chart is appropriate. In fact, the most important differential diagnosis to achieve is to exclude pancreatic carcinoma. Despite non-specific symptoms, imaging findings of thickening of the duodenal wall, cysts within it, smooth CBD stricture when present, normal CBD and pancreatic duct can lead to the correct diagnosis.

## Learning points

1. An uncommon cause of non-specific abdominal pain and vomiting is CDHP.
2. Although CDHP is more common in alcoholic males, it can also be found in non-alcoholic females.
3. On both MDCT and MRI, cystic lesions can be appreciated in the duodenal wall, which is also thickened, and/or soft tissue in the pancreaticoduodenal groove.
4. Even though findings are not specific to diffinitively differentiate groove carcinoma from groove pancreatitis, the simultaneous presence of “patchy”/homogeneous enhancement, normal CBD and duodenal wall cysts is suggestive of this benign condition.
5. Differential diagnosis between groove carcinoma and pancreatitis is crucial as in the latter a pancreas-sparing approach, which seems to be effective and low in morbidity and mortality, could be preferred.

## Consent

Written informed consent for the case to be published (including images, case history and data) was obtained from the patient for publication of this case report.
